# A systematic review of the patient reported outcomes that affect patients with muscle invasive bladder cancer after radical cystectomy and urinary diversion

**DOI:** 10.1002/bco2.339

**Published:** 2024-02-27

**Authors:** John Lahoud, Manish I. Patel, Sayeda Naher, Rebecca Mercieca‐Bebber

**Affiliations:** ^1^ Specialty of Surgery, Sydney Medical School The University of Sydney Sydney NSW Australia; ^2^ Department of Urology Westmead Hospital Westmead NSW Australia; ^3^ NHMRC Clinical Trials Centre, Faculty of Medicine and Health The University of Sydney Sydney Australia

**Keywords:** bladder, cystectomy, patient reported outcome, quality of life, radical cystectomy, urinary diversion

## Abstract

**Objectives:**

To determine the functional domains and symptom scales that affect patients most following radical cystectomy (RC) and urinary diversion (UD), and if a single instrument (or combination) adequately captures these bothersome symptoms. It is unclear whether current patient reported outcome (PRO) instruments that have been used to assess quality of life in patients following RC and UD adequately cover the most bothersome symptoms affecting patients.

**Materials and methods:**

A systematic search of MEDLINE, EMBASE, PubMed, Cinahl and Cochrane was conducted from January 2000 to May 2023 for original articles of patients who had RC and UD since 2000 for muscle invasive bladder cancer. The Preferred Reporting Items for Systematic Reviews and Meta‐Analyses (PRISMA) process was followed. Extracted data included the PRO measures used, domains reported and scores in the first 12 months post‐surgery (short‐term) and after 12 months (long‐term). A conservative threshold of <70 for functional domains and >30 for symptom domains was used to determine which PRO domains were potentially concerning to patients in each study. Quality assessment was performed using the QUALSYST appraisal tool.

**Results:**

Thirty‐five studies met the inclusion criteria, including a total of eight unique PRO instruments. The main findings indicated that physical function was the most concerning PRO for patients with both neobladder (NB) and ileal conduit (IC) in the short and long term. Additionally, bowel, urinary and sexual bother were concerning symptoms for patients with NB in the long‐term, but only in the short‐term for those with IC.

**Conclusions:**

The main issues are adequately addressed using the combination of EORTC QLQ‐C30 and QLQ‐BLM30 instruments.

## 1 INTRODUCTION

Radical cystectomy (RC) with urinary diversions (UDs) such as ileal conduit (IC) or neobladder (NB) formation remains the standard for curative treatment of localised muscle invasive bladder cancer (MIBC). Surgery can be associated with high procedure‐related complications, re‐admission rates and significant psychological distress to the patient.^1^ To optimise clinical outcomes effects on health‐related quality of life (HRQoL) should be considered. Therefore, evaluation of patient reported outcomes (PROs) is an imperative component in quality care.^2^

Several instruments have been designed to better understand and evaluate PRO in patients' post‐surgery. Common instruments used include the European Organisation for Research and Treatment of Cancer Quality of Life Questionnaire (EORTC QLQ‐C30)^3^ accompanied with the Bladder Cancer Muscle Invasive module (EORTC QLQ‐BLM30)^4^; Functional Assessment of Cancer Therapy Bladder Cystectomy (FACT‐Bl‐Cys), formerly known as the FACT‐Vanderbilt Cystectomy Index^5^; Ileal Orthotopic Neobladder–Patient Reported Outcome (IONB‐PRO)^6^, ^7^; and the Short Form Surveys (e.g. SF‐36).^8^

The aim of this systematic review was to evaluate the PRO data following RC and UD for MIBC studies since 2000 to ascertain the aspects of functioning and symptoms that affected patients the most, in the short and long term. Furthermore, our aim was to determine if a single instrument (or combination) adequately captures the symptoms that matter most to patients, and therefore can be used alone to assist patients and clinicians in better understanding the effects of RC and UD on HRQoL.

## 2 MATERIALS AND METHODS

### 2.1 Eligibility criteria

We included full‐text primary research studies published since 2000 that reported PRO data for patients who underwent RC and UD (IC and NB) for localised MIBC. Studies that did not report PRO data by domain or in ways that could be extracted from graphs, tables or text, did not provide separate data for different UD types, including patients with metastatic disease and non‐English language were excluded. This was to enable the description of important outcomes by type of UD. The study was registered with the International Prospective Register of Systematic Reviews (PROSPERO) (ID CRD42021272159).

### 2.2 Information sources

A systematic search of Medline, Embase, PubMed, Web of Science, Cinahl and Cochrane (EBM Reviews) databases was conducted from 1 January 2000 to 5 May 2023. We limited studies to after 2000.

### 2.3 Search strategy

Our search strategy was developed in consultation with an academic librarian and pilot‐tested. The final search terms are listed in Appendix A. Reference lists of relevant studies and journals were also searched.

### 2.4 Selection process

**FIGURE 1:**
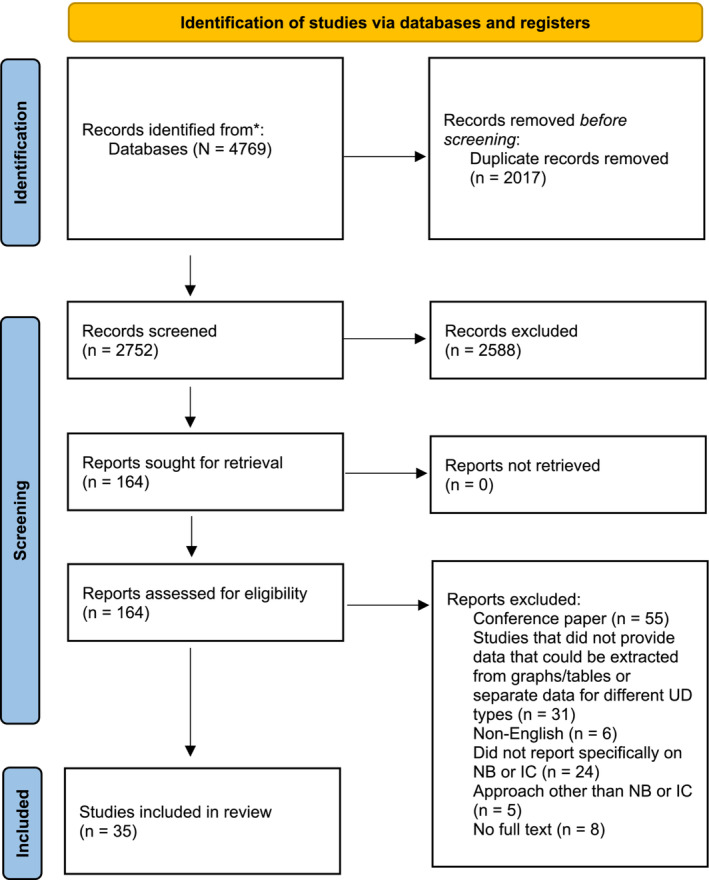
Preferred Reporting Items for Systematic Reviews and Meta‐Analyses flow chart. IC, ileal conduit; NB, neobladder; UD, urinary diversion.

The Preferred Reporting Items for Systematic Reviews and Meta‐Analyses (PRISMA) process was followed (Figure 1). Two reviewers (J.L. and S.N.) independently screened titles and abstracts, and when relevant, assessed full text for eligibility. In the case of a disagreement, mediation by a third author (R.M.B. or M.I.P.) was required (Figure 1).

### 2.5 Data collection process

Study and patient characteristics, type of surgery (open or minimally invasive) and UD, PRO measure, PRO domains assessed, PRO data, adverse events, and functional and symptom scores were all extracted from studies and tabulated (Appendix B).

### 2.6 Study risk of bias assessment

Quality assessment of studies was performed independently by two authors (J.L. and S.N.) using the QUALSYST appraisal tool.^9^ Again, in the case of disagreement, mediation by a third author (R.M.B) was required. We did not exclude any studies based on overall quality or risk of bias as our aim was to review the PRO instruments used and domains assessed rather than to extract any measure of clinical benefit. Rather, we used the scores to assist our overall interpretation of our results.

### 2.7 Synthesis methods

Functional and symptom scales and their respective scores for each instrument were collected from each study and tabulated according to UD type and period of follow‐up (3–12 months; over 12 months, corresponding to ‘short‐term’ and ‘long‐term’ outcomes, respectively) (Table 2). For the ‘long‐term’ assessment, we used the 12‐month assessment data, or the next and closest available time‐point reported (Table 2). We then assessed the clinical importance of data extracted for each domain at the two assessment points. For studies that used the EORTC QLQ‐C30 instrument, thresholds for clinical importance established by Giesinger et al. were used.^10^ The remaining instruments were all standardised on a 0–100 scale, with higher functional scores indicating better functioning and higher symptom scores indicating worse symptoms. None of these remaining scales had formal clinical interpretation guidelines, so we considered functional domain scores below 70 to indicate impaired function, and symptom scale scores above 30 as bothersome symptoms (except in the case of sexual function for EORTC QLQ‐BLM30, where a score below 30 was considered bothersome). Additionally, where individual studies had data contributing to both our timepoints of interest (short‐ and long‐term HRQoL), we compared the two assessments at the domain level to describe the clinical significance of any improvements or regression. We considered 10‐point differences in functional domains and symptom scales to be clinically important.

## 3 RESULTS

### 3.1 Study selection

We identified 4769 records from the literature search for initial assessment. After a full‐text review of 164 articles, 35 articles were included in the systematic review: 25 included patient‐reported outcomes data for IC and 32 for NB (Figure 1).

### 3.2 Study characteristics

Eleven unique PRO instruments were used across the 35 studies (Table 1). The EORTC QLQ‐C30 was the most widely reported instrument (*n* = 13/35, 37% of studies) (Table 1).

**TABLE 1:** Study characteristics and patient demographics.

Characteristic	*N* participants (or studies^a^)
Type of study^a^	
Retrospective	24
Prospective	11
Participants	
Cumulative sample size	4660
Male	3205
Female	800
Not specified^a^	3
Cystectomy	
Open	664
Robotic	376
Not specified^a^	29
Diversion	
Ileal conduit	1730
Neobladder	2184
Not specified^a^	757
Patient‐reported outcome instrument^a^	
QLQ‐C30	13
SF‐36	9
QLQ‐BLM30	8
BCI	5
FACT‐VCI	5
FACT‐G	4
FACT‐BI	4
IONB‐PRO	3

Abbreviations: BCI, Bladder Cancer Index; EORTC‐QLQ‐BLM30, European Organisation for Research and Treatment of Cancer Quality of Life Questionnaire ‐ Bladder Cancer Muscle Invasive; EORTC‐QLQ‐C30, European Organisation for Research and Treatment of Cancer Quality of Life Questionnaire; FACT‐Bl, Functional Assessment of Cancer Therapy ‐ Bladder; FACT‐G, Functional Assessment of Cancer Therapy ‐ General; FACT‐VCI, Functional Assessment of Cancer Therapy ‐ Vanderbilt Cystectomy Index; IONB‐PRO, Ileal Orthotopic Neobladder ‐ Patient Reported Outcomes; SF‐36, Short Form Survey.

^a^ *n* indicates the number of studies.

### 3.3 Risk of bias in studies

Most studies (*n* = 29, 83%) scored above 55% on the QUALSYST appraisal tool, which is considered a liberal threshold for quality.^9^ The range in scores was 31%–91% (Appendix C).

### 3.4 Results of syntheses

#### 3.4.1 Patient reported outcomes of importance

##### Short term (3–12 months) following radical cystectomy with ileal conduit

There were 13 studies with PRO data reported in the first 12 months following IC, which used seven different PRO measures and 24 unique PRO domains (Table 2). Physical, social and emotional functioning were the most frequently reported domains, reported in 11, 10 and 10 of the 13 studies, respectively (Table 2).

**TABLE 2:** Patient reported outcome (PRO) scales that reached clinically relevant thresholds, and studies reporting clinically relevant change in PRO domains over time.

	Ileal Conduit				Neobladder			
	IC < 12 months	IC > 12 months	No. of studies with >10‐point improvement (<12 months–>12 months)	No. of studies with >10‐point decrement (<12 months–>12 months)	NB < 12 months	NB > 12 months	No. of studies with >10‐point improvement (<12 months–>12 months)	No. of studies with >10‐point decrement (<12 months–>12 months)
Number of studies	13	21			18	29		
Functional domains								
Physical	11/11 = 100%	17/22 = 77%	0/8	0/8	12/16 = 75%	20/27 = 74%	0/10	0/10
Social	5/10 = 50%	9/21 = 43%	0/8	1/8	10/16 = 63%	14/28 = 50%	1/11	0/11
Emotional	8/10 = 80%	10/16 = 62.5%	2/7	0/7	10/13 = 77%	15/22 = 68%	1/10	1/10
Functional	5/5 = 100%	7/7 = 100%	0/3	0/3	7/7 = 100%	8/8 = 100%	0/4	0/4
Role	2/4 = 50%	5/13 = 38.5%	1/4	0/4	3/8 = 37.5%	4/16 = 25%	1/5	0/5
Cognitive	1/4 = 25%	1/9 = 11%	1/5	0/5	1/5 = 20%	2/11 = 18%	1/5	0/5
Symptom scales								
Urostomy	0/0	1/3 = 33%	0/0	0/0	1/1 = 100%	1/3 = 33%	1/1	0/1
Mental	0/0	4/4 = 100%	0/0	0/0	1/3 = 33%	5/5 = 100%	0/0	0/0
Sexual	1/4 = 25%	2/5 = 40%	1/2	0/2	4/6 = 67%	4/10 = 40%	2/6	0/6
Daily activities	0/0	0/0	0/0	0/0	1/1 = 100%	2/2 = 100%	1/1	0/1
Bowel function	4/4 = 100%	2/2 = 100%	0/0	0/0	4/4 = 100%	4/4 = 100%	0/3	0/3
Urinary func.	3/4 = 75%	1/3 = 33%	0/0	0/0	4/6 = 67%	2/9 = 22%	0/0	0/0
General health	0/0	4/4 = 100%	0/0	0/0	2/3 = 67%	4/5 = 80%	0/0	0/0
Sleep	0/4 = 0	0/9 = 0	0/4	0/4	1/6 = 17%	4/15 = 27%	1/6	1/6
Pain	0/4 = 0	5/12 = 42%	0/4	0/4	2/8 = 25%	5/16 = 31%	0/5	0/5
Fatigue	2/4 = 50%	2/8 = 25%	0/4	1/4	0/5 = 0	1/10 = 10%	0/5	1/5
Nausea/vom.	2/4 = 50%	4/8 = 50%	0/4	0/4	0/5 = 0	1/9 = 11%	0/5	0/5
Dyspnoea	2/4 = 50%	5/8 = 62.5%	1/4	0/4	2/5 = 40%	4/9 = 44%	0/5	0/5
Appetite loss	0/4 = 0	0/8 = 0	0/4	0/4	0/5 = 0	0/9 = 0	0/5	0/5
Constipation	0/4 = 0	0/8 = 0	0/4	1/4	0/5 = 0	0/9 = 0	0/5	0/5
Diarrhoea	2/4 = 50%	2/8 = 25%	0/4	0/4	2/5 = 40%	1/9 = 11%	0/5	0/5
Fin. impact	3/4 = 75%	4/8 = 50%	0/4	0/4	2/5 = 40%	5/9 = 55%	0/5	0/5
Future worries	0/1 = 0	3/5 = 60%	0/1	0/1	3/3 = 100%	0/8 = 0	0/5	2/5
Abdominal b/f	0/1 = 0	1/5 = 20%	0/1	0/1	0/3 = 0	0/8 = 0	0/3	0/3
Body image	0/2 = 0	3/6 = 50%	0/1	1/1	0/4 = 0	2/9 = 22%	0/4	0/4
Vitality	0/0	4/4 = 100%	0/0	0/0	1/3 = 33%	4/5 = 80%	0/0	0/0
Neobladder	0/0	0/0	0/0	0/0	1/1 = 100%	1/1 = 100%	0/0	0/0
Daily activities	0/0	0/0	0/0	0/0	1/1 = 100%	2/2 = 100%	1/1	0/1
Bowel bother	3/3 = 100%	1/1 = 100%	0/0	0/0	3/3 = 100%	3/3 = 100%	0/2	0/2
Urinary bother	3/3 = 100%	1/1 = 100%	0/0	0/0	3/3 = 100%	3/3 = 100%	0/2	0/2
Sexual bother	3/3 = 100%	1/1 = 100%	0/0	0/0	3/3 = 100%	3/3 = 100%	0/2	0/2

*Note*: Percentages were calculated by taking the number of trials that reported a concerning level of functioning or symptom burden according to predefined thresholds (numerator) as a percentage of the number of trials reporting that domain (denominator).

According to our criteria for patient‐perceived domains of concern or clinical relevance,^10^ physical functioning was a concern in all 11 studies that reported this domain (Table 2). Similarly, functional wellbeing was a concerning domain in all studies for which it was reported (*n* = 5/5), as was bowel functioning (*n* = 4/4), bowel bother (*n* = 3/3), urinary bother (*n* = 3/3) and sexual bother (*n* = 3/3) (Table 2).

##### Long term (greater than 12 months) following radical cystectomy with ileal conduit

There were 21 studies with PRO data reported 12 or more months following IC, which used seven different PRO measures and 28 unique PRO domains (Table 2). Physical functioning/mobility was the most frequently reported domain, bowel function and bother, urinary bother and sexual bother were least commonly reported (Table 2). According to the criteria for patient‐perceived domains of concern or clinical relevance,^10^ consistent with previous, physical functioning remained a domain of most concern with 77% of 22 studies with PRO data reporting concerns (Table 2). General health, mental functioning, functional wellbeing and vitality domains reached concerning levels in all four studies (100%) in which the domains were reported (Table 2).

##### Short term (3–12 months) following radical cystectomy with neobladder

There were 18 studies with PRO data reported in the short term following NB, comprising nine different PRO measures and 30 unique PRO domains (Table 2). Across the 18 studies, there were 16 reported values for physical functioning or mobility, and levels of this domain were deemed concerning in 75% of the 16 instances (Table 2). For the symptom scales, pain met our level for a concerning symptom in *n* = 2/8 (25%) of instances, diarrhoea *n* = 2/5 (40%), financial impact *n* = 2/5 (40%) and dyspnoea *n* = 2/5 (40%). Bowel bother, urinary bother and sexual bother each reached concerning levels in *n* = 3/3 (100%) of studies reporting in the short term (Table 2).

##### Long term (greater than 12 months) following radical cystectomy with neobladder

There were 29 studies that reported PRO data at 12 or more months following NB, which used nine different PRO measures and 29 unique PRO domains (Table 2). Physical functioning was the most reported domain (Table 2).

The physical functioning domain met the criteria for clinical importance in 74% of the 27 studies reported. Of the symptom scales, body image was reported as a concern at 12 months, whereas did not reach concerning levels for any studies that reported short‐term data (Table 2). NB (from Item 17 of the IONB‐PRO instrument ‘living well with NB’) *n* = 1/1 (100%), daily activities *n* = 2/2 (100%), bowel function *n* = 4/4 (100%), bowel bother *n* = 3/3 (100%), urinary bother *n* = 3/3 (100%) and sexual bother *n* = 3/3 (100%) domains reached concerning levels in each study that reported these domains (Table 2).

##### Coverage of patient reported outcomes of importance across patient reported outcome instruments not identified with EORTC QLQ‐C30 and EORTC QLQ‐BLM30 for patients with ileal conduit and neobladder

After reviewing all the symptom and functional domains that reached clinical importance, and after accounting for semantic differences in labelling domains between instruments, the combination of the EORTC QLQ‐C30 and EORTC QLQ‐BLM30 addressed all the issues of importance at both time phases, for both UD types. For IC, 5/5 EORTC QLQ‐C30 and 1/2 EORTC QLQ‐BLM30 domains were considered clinically important in the acute phase, and 8/10 EORTC QLQ‐C30 and 4/6 EORTC QLQ‐BLM30 domains were considered clinically important in the long term. For NB, 5/6 EORTC QLQ‐C30 and 3/3 EORTC QLQ‐BLM30 domains were considered clinically important in the short term, and 10/12 EORTC QLQ‐C30 and 6/8 EORTC QLQ‐BLM30 domains were considered clinically important in the long term.

#### 3.4.2 Change over time

##### Change in patient reported outcomes before and after 12‐month follow up for ileal conduit

Eight studies reported data for both time points for IC (Appendix B). A clinically relevant improvement in emotional,^11^ role^12^ and cognitive^13^ was reported for one study each. Among the studies that had data at both short‐ and long‐term follow‐up, symptom scores were only clinically worse (decrement of greater than 10 points) in one study each for fatigue *n* = 1/4 (25%) studies, constipation *n* = 1/4 (25%) and body image *n* = 1/4 (25%) (Table 2).

##### Change in patient reported outcomes before and after 12‐month follow up for neobladder

Fourteen studies reported data for both time points for NB (Table 2). Across all function domains, a clinically relevant improvement in social,^14^ emotional,^14^ role^12^ and cognitive functioning^13^ was reported for one study each, respectively. Only one study by D'Agostino et al. showed a clinically relevant decrement of greater than 10 points for emotional functioning when the pre‐ and post‐surgery‐12‐month data was compared (Table 2).^14^ One study each reported a greater than 10‐point decrement in future worries, sleep and fatigue.^12^, ^14^ D'Agostino et al. reported data for 171 patients using the IONB‐ PRO, demonstrating a 25‐point improvement in the social domain for patients who have had NB for more than 12 months (Table 2).^14^

### 3.5 Reporting biases

Most studies included in this study reported data from all domains. Only three studies in total did not contain all data for each domain (*n* = 2 for EORTC QLQ‐C30; *n* = 1 EORTC QLQ‐BLM30; *n* = 1 SF‐36) (Table 2).

### 3.6 Certainty of evidence

From the studies that did not include data for all domains of the PRO measure, only Clements et al. reported data for physical and social functioning using the EORTC QLQ‐C30, and sexual function and body image with the EORTC QLQ‐BLM30 however, explored other domains and symptoms scales using other instruments.^15^

## 4 DISCUSSION

### 4.1 Interpretation of results in context of other evidence

This review is the first to identify patient‐reported symptoms and aspects of functioning that have reached concerning levels according to patients with MIBC following UD with IC or NB. The principal findings of this study indicate that physical function was the most concerning PRO for patients with both IC and NB in the short and long term. Additionally, bowel, urinary and sexual bother were concerning symptoms for patients with NB in the long term, but in the short term for those with IC. These issues are covered by using the combination of the EORTC QLQ‐C30 and QLQ‐BLM30 instruments, which collectively address 18 of 28 concerning issues, therefore we recommend this combination in future studies or for use in clinical practice.

To the best of our knowledge, this is the first systematic review to describe patient‐reported functional domains and symptom scales of concern as rated by patients who have undergone IC and NB. Among the studies that assessed physical function in the short and long term, none reported a clinically significant improvement over time, for either UD type (Table 2). A recent prospective study by Abozaid et al. in 2022 used the EORTC QLQ‐C30 and EORTC QLQ‐BLM30 questionnaires and found that physical function remained below baseline in patients with IC up to 12 months post‐RC.^12^ Contrary to this, Tostivinit et al. in 2021 found in a multicentre observational study of 73 patients using the same questionnaires found that physical function for both NB and IC was associated with global health status and quality of life improvement.^16^

Furthermore, for most scales, patients with IC and NB experienced a higher symptom burden, particularly with sexual function and bother. In the context of the current evidence, Clements et al. in 2022 in a single‐centre prospective study demonstrated favourable outcomes in most HRQoL areas except sexual function for IC and NB, and body image for IC.^15^ Whilst our findings have found that sexual function was affected in patients with IC, Goldberg et al. in 2016 found as patients grew older, they were less likely to be bothered by decline in sexual function.^17^

### 4.2 Limitations

Our results are limited by the nature of the data reported. Firstly, studies had varied sample sizes, with some studies having less than 20 patients for analysis.^11^ In some instances, subgroup sizes, for example, gender and operative technique were missing, and therefore, an accurate overall sample size was not completely obtained. Most studies were retrospective (Appendix B). In addition to the presence of heterogenous data within studies utilising the same PRO instrument, the use of diverse measures at different time points made it difficult to compare PRO data directly. Lastly, for scales other than the EORTC QLQ‐C30, there were no guidelines or studies present to determine appropriate clinical thresholds of importance, and therefore, we were required to consider our own.

### 4.3 Implications of results in practice

Our systematic review has demonstrated that the EORTC QLQ‐C30 and EORTC QLQ‐BLM30 effectively capture the symptoms that affect patients most, therefore, future studies can utilise the combination of these two instruments only to ascertain patient HRQoL. This results in more studies reporting data in a similar manner, and thus, future systematic reviews and trials are less likely to be limited by data heterogeneity. Moreover, having less surveys and instruments delivered to patients can place less burden on the patient, lead to less rates of missing data, and produce higher quality data.^18^ Furthermore, the practical implication of our results in clinical practice will assist clinicians in identifying areas of need for supportive care interventions for certain patient subgroups.

### 4.4 Future research

Future studies should aim at assessing the issues that bother patients most in larger cohorts of patients to accurately describe PRO over time, and thus provide clinicians with further insight into ways of optimising patient outcomes throughout the peri‐operative course.

PRO measures are essential in optimising patient outcomes, as they provide both the patient and clinician with insight into issues that can burden patients in the peri‐operative course. Our systematic review has captured the issues that reach clinical thresholds for clinical importance post RC and UD for MIBC. Furthermore, we recommend the EORTC QLQ‐C30 and EORTC QLQ‐BLM30 instruments as a sufficient PRO measure to identify the issues that impact patients most. The combination of these two instruments only for future studies will make it easier to accumulate prospective data and be less burdensome to patients when completing surveys for both clinical and research purposes.

## AUTHOR CONTRIBUTIONS

**John Lahoud:** Study design; data collection; data analysis; draft manuscript; final manuscript write‐up. **Manish Patel:** Study design; review of data; assistance with manuscript write‐up; review of manuscript and feedback; supervision. **Sayeda Naher:** Data collection; assistance with draft manuscript write‐up. **Rebecca Mercieca‐Bebber:** Study design; data analysis; review of data; assistance with manuscript write‐up; review of manuscript and feedback; supervision.

## CONFLICT OF INTEREST STATEMENT

The authors have no interests to declare.

## Appendix A Search strategy for databases.

**MEDLINE (4/5/23)**

**Table:**

#	Search term	Results
1	exp urinary bladder neoplasms/	56 902
2	invasive bladder neoplasm*.tw.	13
3	invasive bladder cancer.tw.	6680
4	invasive bladder carcinoma.tw.	404
5	urinary bladder neoplasm*.tw.	99
6	urinary bladder cancer.tw.	1614
7	urinary bladder carcinoma.tw.	600
8	urological cancer*.tw.	932
9	genitourinary cancer*.tw.	764
10	1 or 2 or 3 or 4 or 5 or 6 or 7 or 8 or 9	60 248
11	quality of life/	218 545
12	patient reported outcome measures/	9156
13	(patient adj1 rated).tw.	2233
14	patient‐reported outcome*.tw.	22 311
15	(patient adj1 report*).tw.	42 115
16	quality of life.tw.	309 167
17	self‐rated.tw.	14 896
18	self‐report*.tw.	172 901
19	QL.tw.	1604
20	QOL.tw.	42 940
21	HRQL.tw.	3699
22	HRQOL.tw.	18 489
23	PROM.tw.	3374
24	PRO.tw.	212 714
25	health status/	85 528
26	health status.tw.	62 745
27	adverse event*.tw.	179 952
28	or/11‐27	1 031 001
29	cystectomy.tw.	15 498
30	urinary diversion.tw.	5591
31	ileal conduit.tw.	2029
32	neobladder.tw.	1784
33	indiana pouch.tw.	168
34	studer bladder.tw.	4
35	urinary conduit.tw.	132
36	urinary stomas.tw.	28
37	Or/29‐37	20 202
38	10 and 28 and 37	877

**EMBASE (4/5/23)**

**Table:**

#	Search term	Results
1	exp urinary bladder neoplasms/	94 933
2	invasive bladder neoplasm*.tw.	17
3	invasive bladder cancer.tw.	11 503
4	invasive bladder carcinoma.tw.	567
5	urinary bladder neoplasm*.tw.	113
6	urinary bladder cancer.tw.	2191
7	urinary bladder carcinoma.tw.	791
8	urological cancer*.tw.	1403
9	genitourinary cancer*.tw.	1199
10	1 or 2 or 3 or 4 or 5 or 6 or 7 or 8 or 9	97 427
11	quality of life/	518 376
12	patient reported outcome measures/	29 906
13	(patient adj1 rated).tw.	3038
14	patient‐reported outcome*.tw.	38 815
15	(patient adj1 report*).tw.	76 345
16	quality of life.tw.	489 368
17	self‐rated.tw.	17 817
18	self‐report*.tw.	227 262
19	QL.tw.	2496
20	QOL.tw.	81 514
21	HRQL.tw.	6313
22	HRQOL.tw.	30 299
23	PROM.tw.	5792
24	PRO.tw.	325 884
25	health status/	136 508
26	health status.tw.	80 812
27	adverse event*.tw.	322 239
28	or/11‐27	1 641 397
29	cystectomy.tw.	25 921
30	urinary diversion.tw.	8303
31	ileal conduit.tw.	3439
32	neobladder.tw.	3065
33	indiana pouch.tw.	261
34	studer bladder.tw.	5
35	urinary conduit.tw.	192
36	urinary stomas.tw.	36
37	Or/29‐37	32 333
38	10 and 28 and 37	1663

**PUBMED (4/5/23)**

(‘urinary bladder neoplasm*’ OR ‘invasive bladder neoplasm*’ OR ‘invasive bladder cancer*’ OR ‘invasive bladder carcinoma*’ OR ‘urinary bladder neoplasm*’ OR ‘urinary bladder cancer*’ OR ‘urinary bladder carcinoma*’ OR ‘urological cancer*’ OR ‘genitourinary cancer*’) AND (‘quality of life’ OR ‘patient reported outcome measur*’ OR [patient near/1 rated] OR ‘patient near report’ OR ‘self‐rated’ OR ‘self‐report’ OR ‘QL’ OR ‘QOL’ OR ‘HRQL’ OR ‘HRQOL’ OR ‘PROM’ OR ‘PRO’ OR ‘health status’ OR ‘adverse event*’) AND (‘cystectomy’ OR ‘urinary diversion’ OR ‘ideal conduit’ OR ‘neobladder’ OR ‘indiana pouch’ OR ‘studer bladder’ OR ‘urinary conduit’ OR ‘urinary stomas’)

**WEB OF SCIENCE (4/5/23)**

(‘urinary bladder neoplasm*’ OR ‘invasive bladder neoplasm*’ OR ‘invasive bladder cancer*’ OR ‘invasive bladder carcinoma*’ OR ‘urinary bladder neoplasm*’ OR ‘urinary bladder cancer*’ OR ‘urinary bladder carcinoma*’ OR ‘urological cancer*’ OR ‘genitourinary cancer*’) AND (‘quality of life’ OR ‘patient reported outcome measur*’ OR [patient near/1 rated] OR ‘patient near report’ OR ‘self‐rated’ OR ‘self‐report’ OR ‘QL’ OR ‘QOL’ OR ‘HRQL’ OR ‘HRQOL’ OR ‘PROM’ OR ‘PRO’ OR ‘health status’ OR ‘adverse event*’) AND (‘cystectomy’ OR ‘rinary diversion’ OR ‘ileal conduit’ OR ‘neobladder’ OR ‘indiana pouch’ OR ‘studer bladder’ OR ‘urinary conduit’ OR ‘urinary stomas’)

**COCHRANE: EBM REVIEWS (4/5/23)**

**Table:**

#	Search term	Results
1	exp urinary bladder neoplasms/	1532
2	invasive bladder neoplasm*.tw.	1
3	invasive bladder cancer.tw.	1116
4	invasive bladder carcinoma.tw.	65
5	urinary bladder neoplasm*.tw.	26
6	urinary bladder cancer.tw.	77
7	urinary bladder carcinoma.tw.	23
8	urological cancer*.tw.	88
9	genitourinary cancer*.tw.	37
10	1 or 2 or 3 or 4 or 5 or 6 or 7 or 8 or 9	2509
11	quality of life/	25 636
12	patient reported outcome measures/	766
13	(patient adj1 rated).tw.	1298
14	patient‐reported outcome*.tw.	9727
15	(patient adj1 report*).tw.	16 996
16	quality of life.tw.	115 580
17	self‐rated.tw.	2564
18	self‐report*.tw.	35 634
19	QL.tw.	569
20	QOL.tw.	21 318
21	HRQL.tw.	1273
22	HRQOL.tw.	6336
23	PROM.tw.	807
24	PRO.tw.	14 353
25	health status/	3822
26	health status.tw.	10 470
27	adverse event*.tw.	126 681
28	or/11‐27	285 203
29	cystectomy.tw.	1533
30	urinary diversion.tw.	270
31	ileal conduit.tw.	77
32	neobladder.tw.	115
33	indiana pouch.tw.	1
34	studer bladder.tw.	0
35	urinary conduit.tw.	3
36	urinary stomas.tw.	0
37	Or/29‐37	1636
38	10 and 28 and 37	155

**CINAHL (17/8/2021)**

## Appendix B Characteristics of the included studies.

**Table:**

Lead author	Year published	Research location (country)	Number of centres	Research period (start date)	End date	Data	Study design
Abozaid	2021	UK	1	2016	2017	Prospective	Cohort
Arman	2020	Armenia	1	2016	2018	Prospective	Cross sectional
Autorino	2009	Italy	5	2002	2007	Retrospective	Cross sectional
Becerra	2020	USA	15	2011	2014	Prospective	RCT
Cerruto	2017	Italy	5	2010	2013	Retrospective	Cross sectional
Chabowski	2018	Poland	1	2015	2015	Retrospective	Cohort
Clements	2022	USA	1	2008	2014	Prospective	Cohort
D'Agostino	2016	Italy	5	2006	2011	Retrospective	Cross sectional
Dey	2019	India	1	2015	2017	Prospective	Cohort
Erber	2012	Germany	1	1993	2007	Retrospective	Cohort
Fujisawa	2000	Japan	1	2000	2000	Retrospective	Cross sectional
Gellhaus	2017	USA	1	1991	2009	Retrospective	Cross sectional
Gilbert	2007	USA	1	1995	2004	Prospective	Cross sectional
Harano	2007	Japan	1	1992	2003	Retrospective	Cross sectional
Hardt	2000	Germany	1	1996	1997	Prospective	Cohort
Imbimbo	2015	Italy	5	2010	2013	Retrospective	Cross sectional
Kikuchi	2006	Japan	1	1987	2002	Retrospective	Cross sectional
Kretschmer	2017	Germany	1	2013	2014	Retrospective	Cross sectional
Kretschmer	2020	Germany	1	2014	2015	Retrospective	Cross sectional
Large	2014	USA	1	2011	2012	Prospective	Cross sectional
Mansson	2007	Sweden	3	2007	2007	Prospective	Cross sectional
Mansson	2002	Sweden	1	1987	2000	Retrospective	Cross sectional
McGuire	2000	USA	1	1989	1997	Retrospective	Cohort
Moeen	2018	Egypt	1	2011	2016	Prospective	Cross sectional
Moncrief	2017	USA	1	2009	2014	Retrospective	Cross sectional
Osawa	2021	Japan	7	1999	2017	Retrospective	Cross sectional
Rouanne	2014	France	1	1995	2011	Retrospective	Cross sectional
Satkunasivam	2016	USA	1	2012	2013	Retrospective	Cross sectional
Singh	2014	India	1	2007	2012	Prospective	Cross sectional
Siracusano	2019	Italy	6	2007	2013	Retrospective	Cross sectional
Sogni	2008	Italy	3	2000	2004	Retrospective	Cross sectional
Stenzelius	2016	Sweden	1	2014	2014	Retrospective	Cross sectional
Yang	2013	China	1	2007	2009	Retrospective	Cross sectional
Yoneda	2005	Japan	1	1996	2003	Retrospective	Cross sectional
Zahran	2014	Egypt	1	2011	2012	Retrospective	Cohort

## Appendix C Overall quality assessment of studies using the QUALSYST appraisal tool.

**Table:**

Record number	Reference (year)	Total score	Total percentage
1	Abozaid 2021	14	63.6
2	Arman 2020	13	59.1
3	Autorino 2009	12	54.5
4	Becerra 2020	21	87.5
5	Cerruto 2017	16	72.7
6	Clements 2022	19	79.2
7	Chabowski 2018	8	36.4
8	D'Agostino 2016	13	59.1
9	Dey 2019	10	45.5
10	Erber 2012	10	45.5
11	Fujisawa 2000	7	31.8
12	Gellhaus 2017	10	45.5
13	Gilbert 2007	14	63.6
14	Harano 2007	13	59.1
15	Hardt 2000	17	77.3
16	Imbimbo 2015	17	77.3
17	Kikuchi 2006	13	59.1
18	Kretschmer 2017	13	59.1
19	Kretschmer 2020	13	59.1
20	Large 2014	15	68.2
21	Mansson 2007	14	63.6
22	Mansson 2002	17	77.3
23	McGuire 2000	12	54.5
24	Moeen 2018	12	54.5
25	Moncrief 2017	16	72.7
26	Osawa 2021	16	72.7
27	Rouanne 2014	16	72.7
28	Satkunasivam 2016	18	81.8
29	Singh 2014	20	90.9
30	Siracusano 2019	18	81.8
31	Sogni 2008	19	86.4
32	Stenzelius 2016	14	63.6
33	Yang 2013	14	63.6
34	Yoneda 2005	18	81.8
35	Zahran 2014	18	81.8
